# Scope, quality, and inclusivity of clinical guidelines produced early in the covid-19 pandemic: rapid review

**DOI:** 10.1136/bmj.m2371

**Published:** 2020-06-12

In this research article by Dagens and colleagues (BMJ 2020;369:m1936, doi:10.1136/bmj.m1936, published 26 May 2020), co-author Vincent Cheng’s surname was misspelt. The online article and PDF will be updated in due course.

